# Candida Colonization on the Denture of Diabetic and Non-diabetic Patients

**Published:** 2009

**Authors:** Mohammad Hossein Lotfi-Kamran, Abbas Ali Jafari, Abbas Falah-Tafti, Ehsan Tavakoli, Mohammad Hossein Falahzadeh

**Affiliations:** * Assistant Professor, Department of Prosthodontics, School of Dentistry, Yazd University of Medical Sciences, Yazd, Iran; ** Associate Professor, Department of Parasitology and Mycology, School of Medicine, Yazd University of Medical Sciences, Yazd, Iran; *** Dentist, Yazd University of Medical Sciences, Yazd, Iran; **** Assistant Professor, Department of Bioinformatics, School of Health, Yazd University of Medical Sciences, Yazd, Iran

**Keywords:** Candida albicans, Colonization, Denture, Diabetes

## Abstract

**Background:**

Oral candidiasis is a common opportunistic infection in diabetic patients. Presence of denture in the oral cavity of diabetic patients can promote *Candida* colonization and results in the higher incidence of oral and systemic candidiasis. The general purpose of the present study was to evaluate and compare *Candida* colonization in denture of diabetic patients and non-diabetic control group.

**Methods:**

In current case-control study, samples for mycological examinations were collected from the palatal impression surface of maxillary dentures from 92 edentulous patients including 46 dia-betic and 46 non-diabetic denture wearers. All samples were cultured directly on sabouraud agar me-dium and isolated colonies were counted and identified based on specific tests. Data were statistically analyzed using Mann-Whitney and Spearman correlation tests.

**Results:**

The higher density of isolated colonies was seen in diabetic group in compare with control group (P = 0.0001). There was a statistically significant correlation between the blood glucose level (P = 0.0001) and the duration of denture usage (P = 0.022) with the colonization of *Candida* on denture of diabetic patients. *C. albicans* was the most common isolated Candida species in both groups, though diabetic patients with dentures had more non-albicans *Candida* isolated from their dentures compared to non-diabetic patients.

**Conclusions:**

Mycological findings from the present study revealed that diabetes mellitus can in-crease colonization of *Candida* in denture and mouth. By elimination of local and systemic factors in diabetic patients and improving their oral health care, *Candida* colonization and the risk of oral and systemic candidiasis will be decreased.

## Introduction

Diabetes mellitus is a common and growing global health problem which causes several complica-tions. Periodontal diseases are considered the sixth complication of this disease. Diabetics have an increased predisposition to the manifestations of oral diseases like candidiasis, which is associated with poor glycemic control and therapeutic dentures.^1^ This predisposition also contributes to xerostomia, which may be due to increased glucose levels in oral fluids or immune dysregulation.^2^ Wearing complete denture is also known as an-other risk factor, which can promote colonization of *Candida*, produce Candidal biofilm and result in oral candidiasis. Association of denture and diabetes can increase the incidence of oral *Candida* dis-orders in diabetic patients. *Candida* species are present in the oral cavity of almost half of the population without causing disease.^2^ Asymptomatic carriage may cause a higher risk of *Candida* associated complications through yeast infections if they become immunosuppressed.^3^,^4^ Diabetes mellitus is a chronic metabolic disease, which causes several disorders. Immunodeficiency and increased susceptibility to opportunistic infections are seen in diabetic patients. Colonization of Candida is more prevalent in people with diabetes mellitus^5^–^7^ and many studies have shown a higher prevalence of *Candida* colonization in the oral cav-ity of diabetics compared with non-diabetic individuals.^1^,^8^ In addition, significantly higher preva-lence of oral candidiasis in people with diabetes is reported^4^. Also *Candida* infection is found commonly in denture wearers. Acrylic dentures are an important predisposing factor for oral candidosis as these appliances, which are usually ill fitting with suboptimal hygiene, act as reservoirs of infection. For instance, high salivary yeast counts are much more common in complete denture wearers than in dentate individuals.^8^ Commensal existence of intra-oral *Candida* species varies from 20% to 50% in a healthy edentulous population and up to 75% in denture-wearers.^9^ The manifestation of oral candidiasis can occur in many different forms includ-ing median rhomboid glossitis, atrophic glossitis, denture stomatitis (stomatitis prothetica), and an-gular cheilitis. Usually, oral candidosis is associated with a high density of yeasts in the lesions.^10^,^11^ Oral candidosis have been reported in 9% to 65% of the population.^12^,^13^ These variations are far too important to be explained by demographic varia-tions or socio-economic dissimilarities alone, but may be linked, in part, to differences in denture usage and hygiene habits as well as to underlying systemic predisposing factors.^14^ The aim of the present study was to assess the prevalence of yeast on the denture of diabetic and non-diabetic denture wearers.

## Materials and Methods

Forty six non-insulin dependent diabetes patients (23 men and 23 women), who were complete denture-wearers and were admitted to the Yazd Centre of Diabetes Research were entered consecutively into this study as case group. The patients were in the age range of 62.8 ± 11.2 years. Their diabetic status was determined by history of previous diag-nosis of diabetes, and their blood glucose levels were determined before sample collection using IME-DC (Germany) glucometer (with the mean fasting blood glucose level of 289.2 ± 72.8 mg/dl). The control group included 46 (24 men and 22 women) non-diabetics (with the fasting blood glu-cose level less than 110 mg/dl) selected from pa-tients attending the Department of Prosthetic Dentistry, University of Yazd Medical Sciences and Health Services.

Each patient completed a medical and dental history and signed an informed consent document. All patients accepted denture sample collection. Samples were obtained in the morning when pa-tients fasted by swabbing from the palatal impres-sion surface of maxillary dentures, and cultured on sabouraud glucose agar plates. All isolated yeasts were first counted and then identified by sub-culturing on CHROMagar *Candida* (CHRO-Magar, France), performing the germ tube test, hyphae/pseudohyphae and chlamydospores growth as described by Terai and Sandven.^15^,^16^ Each isolated colony was known as a colony forming units on denture in this study as used in reference method.^17^

### 

#### Inclusion and exclusion criteria

All patients in both groups had worn complete dentures for more than one year. No patient in either group had received antibiotics, steroids or immune therapy, or used any antiseptic mouth wash for the 6 months before entering the study. People with complete denture with more than one year fasting blood glucose (FBS) level more than 130 mg/dl (in test group), and people without any diabetic history and FBS less than 110 mg/dl (in control group) were included in this study.

#### Statistical analysis

Mann-Whitney test was used to analyze the differ-ences between the means of isolated colonies (dis-tribution of data was not normal). Spearman corre-lation test was used for analyzing the correlation between serum glucose level and duration of denture usage with the *Candida* colonization on denture. Differences were considered significant if P < 0.05. All statistical calculations were per-formed using SPSS software.

## Results

Positive culture of *Candida* enrolled on the denture of all diabetic subjects but this was seen in 78.3% of non-diabetics. Participants’ demographic charac-teristics are summarized in table 1. The control group included more men. A significantly greater proportion of subjects with diabetes mellitus had higher colonization of *Candida* compared to con-trol subjects. The average number of isolated colonies was 296.9 in diabetics, though it was 64.7 in non-diabetic group; and Mann-Whitney test showed that this difference was statistically sig-nificant (P = 0.0001).

**Table 1:** Mean (SD) of variations for denture *Candida* colonization.

Variables	Diabetic	Non-diabetic	P-value
Age (years)	62.8(11.2)	61.4(12.4)	0.7010
FBS (mg/dl)	289.2(72.8)	94.7(8.2)	0.0001
Denture use (years)	5.1(2.04)	5.2(1.8)	0.2410
Colony counts (CFU/each sample)	296.9(136.6)	64.7(42.6)	0.0001

**Figure 1:**
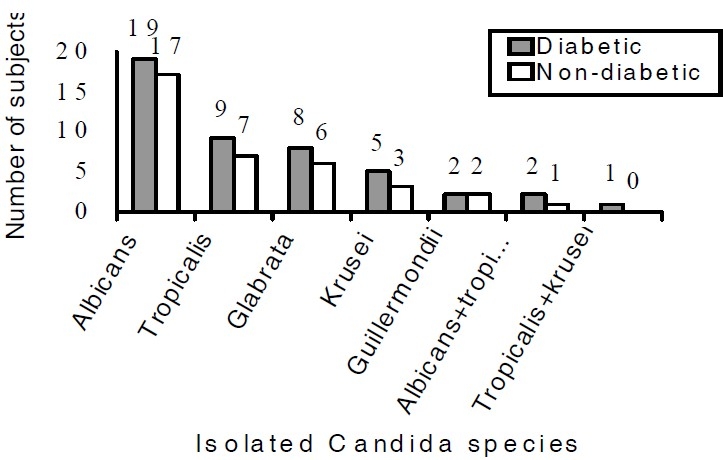
Frequency of Candida species isolated from denture of diabetic and non-diabetic groups.

*C. albicans* was the most commonly isolated species in both groups followed by *C. tropicalis*, *T. glabrata*, *C. krusei*, and *C. guillermondii* (figure 1). The higher isolation and colonization of non-*albicans* species was seen in diabetic compared to non-diabetic group (P ≤ 0.05). A statistically sig-nificant correlation was found between the number of colonies and amount of serum glucose level in diabetic group (P = 0.0001). Also, there was seen a statistical significant correlation between the colo-nization rate and the duration of using denture in diabetic group (P = 0.022).

## Discussion

Candidal infections are a major problem in the world, especially among the immunosuppressed people.^7^,^10^,^11^,^18^ Furthermore, increased susceptibil-ity to periodontal^19^ and oral infections with *Candida* spp. has long been associated with diabetes mellitus, but the results remain controversial and contradictory.^1^,^4^,^5^,^18^ In the present study a higher colonization of *Candida* species was seen in diabetic patients compared with control group. This finding showed statistical significance when compared with non-diabetic patients (P = 0.0001). A similar trend was observed by some investigators that Candidal carriage was higher among diabetics wearing dentures.^20^–^22^ However, in Daniluk’s study^13^ there was no statistically significant differ-ence between the two groups in *Candida albicans* colonization. Most of similar studies reported iso-lated *Candida* species as *C. albicans* and other *Candida* species; however in current study, differ-ent *Candida* species were also identified based on the specific diagnostic tests, which is an advantage for this study. In our study, the frequency of oral non-albicans *Candida* isolates was more common in diabetic patients with denture than non-diabetic patients with denture (P = 0.0207).

A statistically significant correlation was found between the number of colonies and the serum glu-cose levels in diabetic group (P = 0.0001). Also there was a statistically significant correlation between the colonization rate and the duration of us-ing denture in diabetic group (P = 0.022). *Candida* colonization in denture wearers, especially immunocompromised patients, can be disruptive to dental treatment and may be a barrier to patients’ health. The surface irregularities of acrylic resin are a factor in the entrapment of microorganisms, especially *Candida albicans*. Consequently, con-trolling the spread of fungal infection in risk pa-tients who wear removable prostheses and who are more susceptible to fungal infections because of their immunosuppression is of critical importance.

The higher colonization of non-*albicans Candida* species in diabetic group in the present study supported other reports by different studies^20^,^23^ and showed diabetics’ higher susceptibility to colonization with these species. Unfortunately, these species are less susceptible to common anti-fungal drugs than *C. albicans*.^24^ This fact high-lights the importance of controlling the oral colo-nization in diabetic group. *C. dubliniensis* is a new species of *Candida*, which was isolated from the diabetic oral cavity in few similar studies^23^ but this species wasn’t identified in the present study.

Concerning the maintenance of denture hygiene in order to improve oral mucosal health, the participants were informed to control their blood glucose level, and to regularly clean their dentures and keep them dry overnight. These are simple and efficient ways to control yeast colonization in denture wearers.^25^,^26^ This precaution seems appropriate since it is apparently difficult to improve the oral hygiene in denture wearers by mechanical or chemical plaque control.^27^,^28^

## Conclusion

Since using complete denture causes more susceptibility to *Candida*l colonization, oral candidiasis and other consequences particularly in diabetic patients, controlling the level of serum glucose, drinking enough water in diabetic patients with xerostomia, regularly disinfection of denture and leaving the dentures exposed to the air at night are the possible solutions for improving the diabetic oral health status.
